# Supplementary material for: SIN-Like Pathway Kinases Regulate the End of Mitosis in the Methylotrophic Yeast Ogataea polymorpha
Source: Cells. 2022 Apr 30;11(9):1519. doi: 10.3390/cells11091519 (PMC9105162; doi:10.3390/cells11091519)
Supplement: Supplementary file 1 [file cells-11-01519-s001.zip › cells-1681889-supplementary.pdf]

Figure S1

A

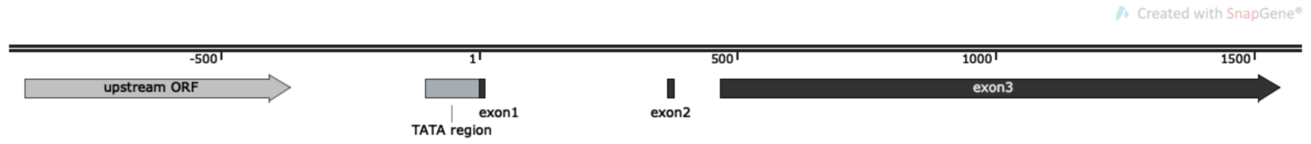

B

```
                                      Upstream ORF
-900 AAATTTATCTACATCTGTTATGGCTGGAGCTTCCGACAAAAAGCAGGCCGCGGCCAATTCTTCTATCTTGAAGCAGCTGCATCTTGCCTCTGTCGTGGTG
-800 AACGTGGTGGCTCTACTTGCGCTTTTTGTTTTGCACAGGCCCGCAGCTAAAAAATACTACTTTATATTCAGTCTCCCGGGCTTGGGCTGTCAGTATGTGC
-700 TCGAAAAGGTGGGCAGGCCCAAATACCACATGAATCCTCAGGGATACAGCGTTCTATCTTCTGCTGGACAGGATCTACAACAGCAGGGTCTCACAGAGTA
-600 CATGATCGACATTGTTTATTTGACACTGGCAATTGACGTTTTGATGGTCCTTTTCGGTTCCAACAAGGCCTGGCTGCTTCTGCTGGCTGTGCCTAGCTAC
-500 GCTGGATACAAATTGAAGGGTTTCATTTCGCCATTCTTTGCCCGCAGGCAGAAAGAAGAGCAAATAGAGACAGAGCCCGTGAAAAGCAAGCGGCAGCAAA
-400 AGTTGGAGAGCAGGAAGACCAAGGTCAGATACAGGTGAGAGGAACACGTGTGAGTGTGGGTCCGAGACCATCTCAGCAGCACAGCCGTTTAGATTACAAA
-300 TTATTTAGCGGAAGCCAGGTAAATGGTGCAAGTGTAGCCAATGCAATGCTCTCCATCACACGCAGTGCACACGAGATTCGGTTCCGGTGTACAGATGTGA
-200 CAATATTTTTTCCGGACGTCACGTTACGAGCCCGCAAAGCCCCTTCCGGTGGTCGCGAATATTCCAATCAGCAGGCTCCAGCAGATCGCCTCCATCTTGT
-100 TGAGTTGTCCTCTCAGTTTTTATAAAAGTTTTTATTTTACCTTGTTTTGTAAATTAGATCTTTCAATTGGTTGGAATCATCTTAATTGAGTTATAGCAAA
  +1 ATGGACGGAGGTAAGTACTGAGGAAATTCGGTTCCGGGCCCGATTGAGATCAGTGGCAAAGGAGCGGGCAATTCCCAAAATTGTTTGGAGGTCAAATGCC
+100 AAAGCGCGGATTCTTGCAGCCCAGACAGTCAGCGTGGCGTTGTGGTATCACGCCTGCGAGTAGGTGGAGGACAGAGCGATAGGATGCGGCATGCTAAGGC
+200 AGTATTGGTGACGATAATTGTCTTTTTCAATGCAGCACAATTCGCCTCAGTCCGAAAAAAAACAACCAGACGTGTTTGGTTGCCCTTACTCGACCGATCC
+300 AGCCGCAAAGTGGAGATTGGGGCCCGGAAGTCGACGGTTTACATGTTTCTAACACTTTTATAGAAGACGTTGCTGCTGTAAGTACTAAGCGGTCCATATA
+400 CGAGGGTTGAAAAGTTAAGCCCGAGTTGCGCACCGCATATGCGAACCCAAAGTTACTAACAATTTAGTTGGTCATTGATAACGGATCCGGTATGTGCAAA
+500 GCCGGCTTTGCTGGTGACGACGCTCCAAGAGCCGTGTTTCCTTCGATTGTCGGAAGACCTAGACACCAGGGTATCATGGTCGGTATGGGTCAAAAGGACT
+600 CCTACGTTGGAGACGAGGCCCAATCCAAGAGAGGTATCTTGACTCTGAGATACCCAATTGAGCACGGTATCGTTACCAACTGGGACGACATGGAGAAGAT
+700 CTGGCACCACACTTTCTACAACGAGTTGAGAGTTGCGCCAGAAGAGCACCCAGTTTTGCTGACTGAGGCTCCAATGAACCCTAAGTCCAACAGAGAAAAG
+800 ATGACCCAGATTTTGTTCGAGACGTTCAACGTGCCAGCATTCTACGTTTCGATCCAGGCTGTGCTGTCGTTGTACTCGTCTGGTAGAACGACCGGTATTG
+900 TCTTGGACTCTGGTGACGGTGTTACGCACGTTGTTCCAATCTATGCTGGTTTCTCTCTCCCACACGCCATTTTGAGAATCGACTTGGCCGGTAGAGACTT
+1000 GACTGACTACTTGATGAAGATTCTTTCTGAGAGAGGTTACACGTTCTCCACCACTGCCGAAAGAGAAATTGTCAGAGACATCAAGGAGAAGCTCTGTTAC
+1100 GTGGCTCTTGACTTTGACCAGGAGCTGCAGACTTCTTCCCAGTCTTCTGCTATCGAGAAGTCCTATGAGTTGCCTGATGGACAGGTGATCACCATCGGTA
+1200 ATGAGAGATTCAGAGCCCCAGAAGTTCTGTTCCACCCAGGTCCGCTTGGACTGGAAGCCGCTGGTATTGACCAAACCACTTACAACTCCATCATCAAGTG
+1300 TGATGTCGATGTTAGAAAGGAGCTGTACGGTAACATTGTCATGTCTGGTGGTACTACCATGTTCCCAGGTATTGCTGAGAGAATGCAAAAGGAGATCACT
+1400 GCCTTGGCACCATCTTCGATGAAGGTCAAGATCATTGCTCCACCGGAGAGAAAGTACTCTGTTTGGATCGGTGGTTCCATCTTGGCCTCCTTGTCCACTT
+1500 TCCAACAGATGTGGATTTCCAAGCAAGAATACGACGAGTCCGGACCATCCATTGTCCACCACAAGTGTTTCTGATTCTGTCGGGTCTATTCTTGCGCCTG
```

Figure S1. Promoter region of *OpACT1* gene.

(A) Schematic map of the DNA sequence surrounding *OpACT1* gene (scaffold_5:1,140,451..1,142,929) drawn with SnapGene (GSL Biotech LLC). The DNA sequence was obtained at the genome portal of the Department of Energy Joint Genome Institute (https://mycocosm.jgi.doe.gov/Hanpo2/Hanpo2.home.html) [6]

(B) Genomic nucleotide sequence of *OpACT1* gene and the upstream region. Dark grey boxes with bold letters are exons 1-3. Itallics in a grey box is the TATA-like sequence used in the TetO7 promoter for *O. polymorpha.* Light grey marks the upstream ORF.

Figure S2

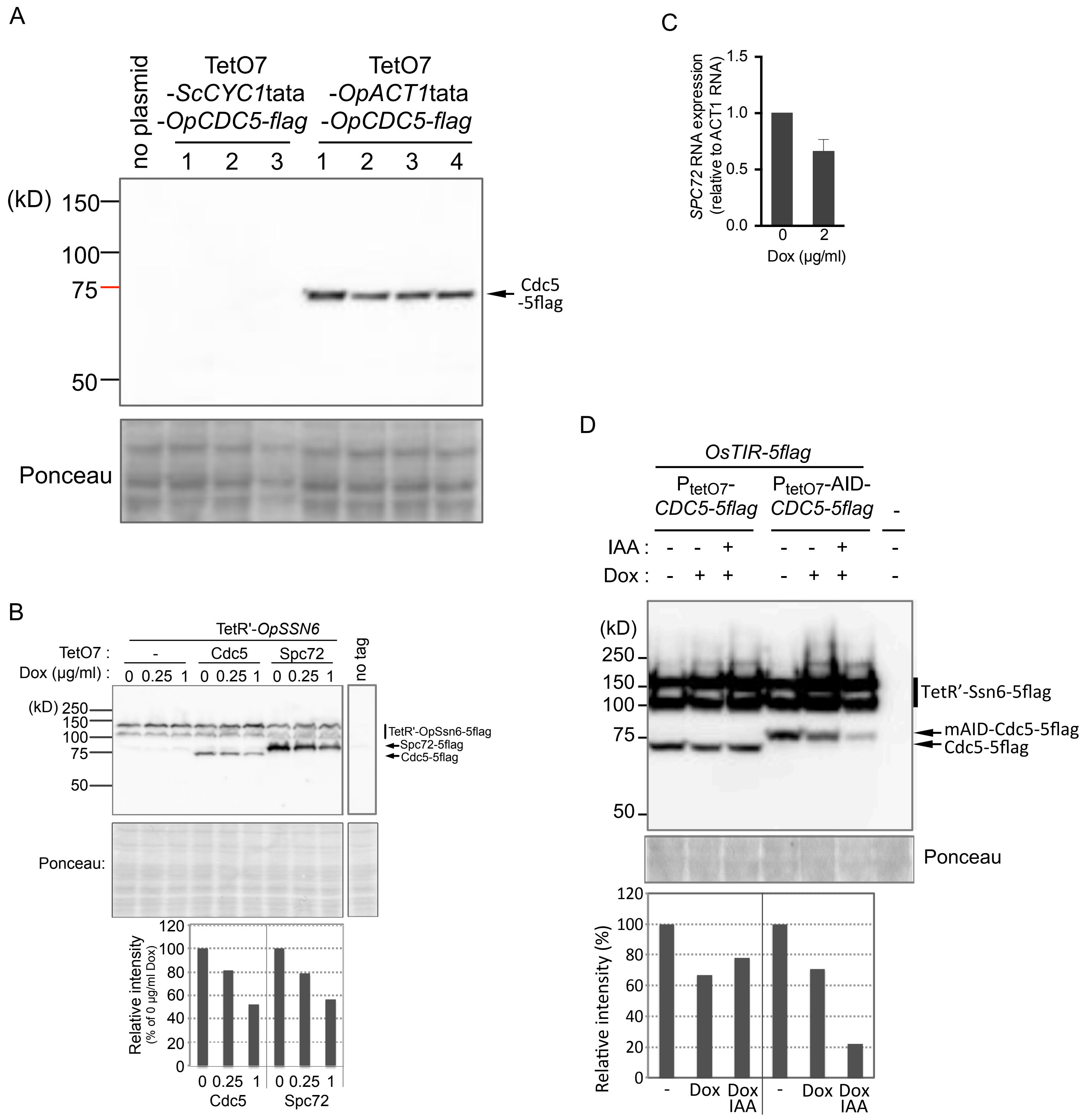

A
no plasmid
TetO7
-ScCYC1tata
-OpCDC5-flag
1 2 3
TetO7
-OpACT1tata
-OpCDC5-flag
1 2 3 4
(kD) 150
100
75
50
Cdc5
-5flag
Ponceau
B
TetR'-OpSSN6
TetO7 :
-
Cdc5
Spc72
Dox (μg/ml) :
0 0.25 1 0 0.25 1 0 0.25 1
no tag
(kD) 250
150
100
75
50
TetR'-OpSsn6-5flag
Spc72-5flag
Cdc5-5flag
Ponceau:
Relative intensity
(% of 0 μg/ml Dox)
120
100
80
60
40
20
0
0 0.25 1 0 0.25 1
Cdc5
Spc72
C
SPC72 RNA expression
(relative to ACT1 RNA)
1.5
1.0
0.5
0.0
0 2
Dox (μg/ml)
D
OsTIR-5flag
PtetO7-
CDC5-5flag
PtetO7-AID-
CDC5-5flag
-
IAA : - - + - - + -
Dox : - + + - + + -
(kD)
250
150
100
75
50
TetR'-Ssn6-5flag
mAID-Cdc5-5flag
Cdc5-5flag
Ponceau
Relative intensity (%)
120
100
80
60
40
20
0
- Dox Dox IAA - Dox Dox IAA

Figure S2 The iAID system in *O. polymorpha*

(A) Cdc5 protein levels expressed from the modified tetracycline-responsive promoters. Cdc5-5flag protein was exogenously expressed from TetO7-$TATA_{ScCYC1}$ promoter or TetO7-$TATA_{OpACT1}$ promoter in wild type cells expressing TetR-VP16. Total cell extracts were prepared and subjected to a western analysis using anti-flag antibody. Three and four independent clones of HPH1942 and HPH1943, respectively, were analysed. No plasmid: wild type cells not carrying the *OpCDC5-5flag* plasmid (HPH656). Cdc5-5flag protein was not detected when expressed from TetO7-$TATA_{ScCYC1}$ promoter.

(B) Cdc5-5flag, Spc72-5flag, or GFP-5flag protein level expressed from the TetO7 promoter in the presence of doxycycline. CDC5-5flag (HPH2004), SPC72-5flag (HPH2008), or GFP-5flag (HPH2009) genes were placed under TetO7-$TATA_{OpACT1}$ promoter in wild type cells carrying P_{CMV}-tTA, P_{OpTEF1}-*TetR'-OpSSN6-5flag*. Cells were pre-cultured in YPDS at 30 °C, then doxycycline was added at the indicated concentration and incubated for 5 h at 30 °C. Total cell extracts were prepared and subjected to a western analysis using anti-flag antibody. Note that the two slowest migrating bands correspond to the TetR'-Ssn6-5flag. No-tag: wild type (HPH951). Intensity of bands was quantified with ImageJ software.

(C) The *SPC72* RNA expression level. Wild type cells carrying P_{TetO7}-*OpSPC72-5flag*, tTA, and P_{OpTEF1}-*TetR'-OpSSN6-5flag* (HPH2008) were grown in YPDS. Doxycycline was added at indicated concentration and cells were incubated at 30 °C for 7 h. Total RNAs were subjected to qRT-PCR analysis for *SPC72* RNA and *ACT1* RNA. Relative expression to that without doxycycline was calculated. The experiment was performed in triplicate and the combined results are shown.

(D) Cdc5 protein level expressed by the $iAID^{Op}$ system. Wild type diploid cells carrying either P_{TetO7}-*CDC5-5flag* (HPH2044) or P_{TetO7}-*mAID-CDC5-5flag* (HPH2046) as well as P_{ADH1}-*OsTIR*, P_{CMV}-tTA, P_{OpTEF1}-*TetR'-OpSSN6-5flag* and wild type haploid cells were grown in SD medium supplemented with appropriate nucleotide and amino acids in the presence or absence of 20 mg/mL doxycycline. IAA and 1NM-PP1 were added at 0.5 mM and incubated at 30 °C for 2.5 h. Total cell extracts were prepared and subjected to a western analysis using anti-flag antibody. Intensity of bands was quantified with ImageJ software. Similar result was shown in Fig. S4.

Figure S3

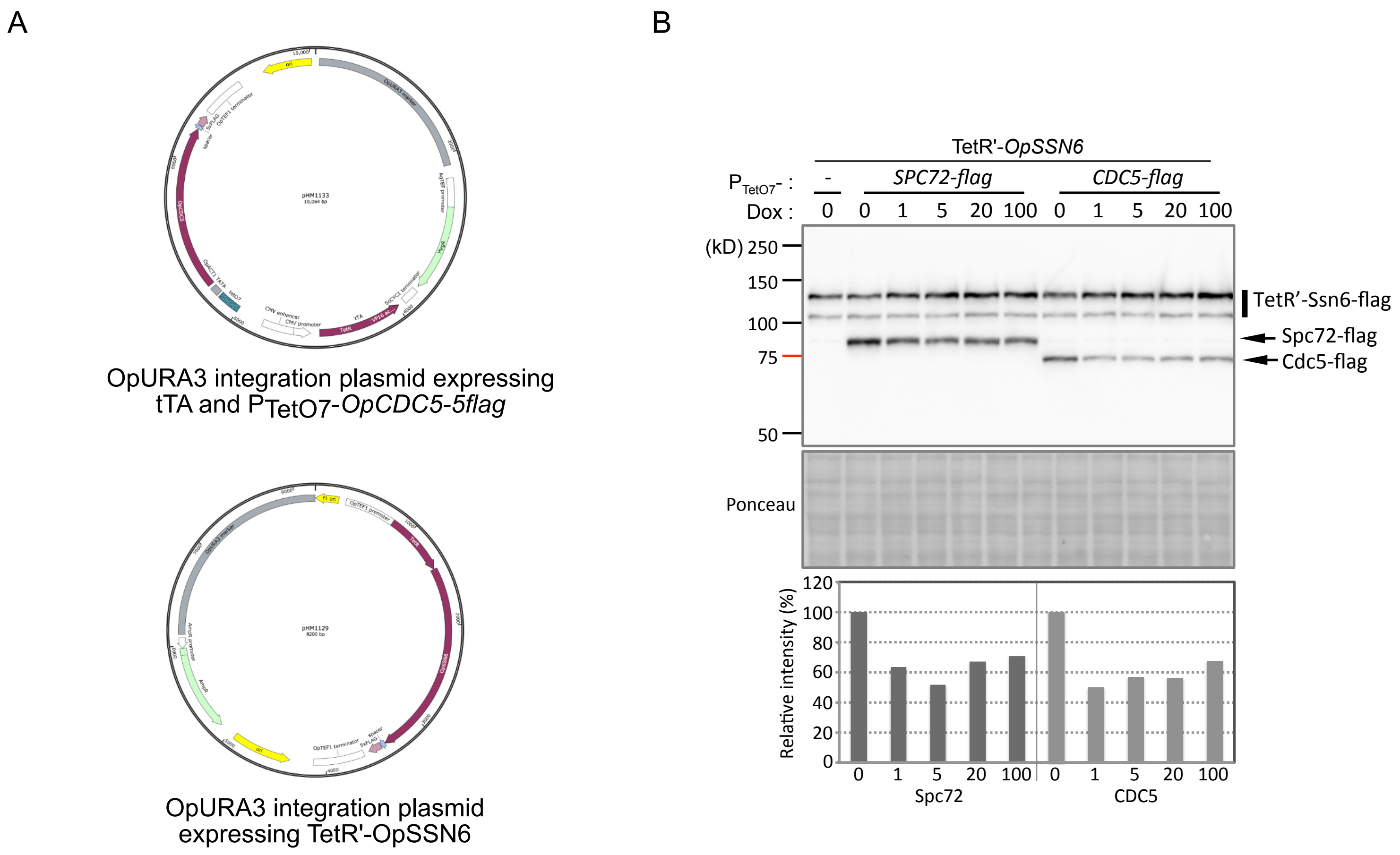

Figure S3 Tet-OFF system in *O. polymorpha*

(A) Schematic maps of the plasmids used to construct the *OpCDC5* Tet-OFF strain. Both pHM1129 and pHM1133 plasmids were integrated into *OpURA3* locus. The maps were drawn with SnapGene.

(B) Cdc5-5flag and Spc72-5flag protein level expressed from the TetO7 promoter in the presence of doxycycline. *CDC5-5flag* (HPH2004) and *SPC72-5flag* (HPH2008) genes were placed under the TetO7-TATA$_{OpACT1}$ promoter in wild type cells carrying P$_{CMV}$-tTA, P$_{OpTEF1}$-*TetR'-OpSSN6-5flag*. Cells were pre-cultured in YPDS at 30 °C, then doxycycline was added at the indicated concentration and incubated for 5 h at 30 °C. Total cell extracts were prepared and subjected to a western analysis using anti-flag antibody. Wild type cells carrying P$_{OpTEF1}$-*TetR'-OpSSN6-5flag* (HPH1926) was used as the negative control. Note that the two slowest migrating bands correspond to the TetR'-Ssn6-5flag. Intensity of bands was measured with ImageJ. Addition of doxycycline efficiently lowered the level of OpCDC5-flag and OpSPC72-flag proteins.

Figure S4

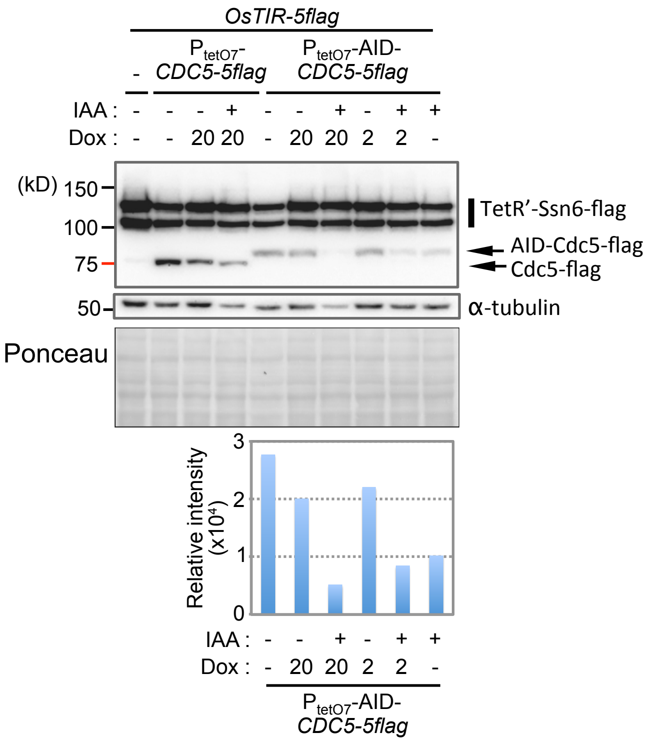

Figure S4 Regulation of *CDC5-5flag* expression by the iAID^{Op} system

Cdc5 protein level expressed by the iAID^{Op} system. Wild type diploid cells carrying either P$_{TetO7}$-*CDC5-5flag* (HPH2049) or P$_{TetO7}$-*mAID-CDC5-5flag* (HPH2053) as well as P$_{ADH1}$-*OsTIR*, P$_{CMV}$-tTA, P$_{OpTEF1}$-*TetR'-OpSSN6-5flag* and wild type diploid cells carrying P$_{ADH1}$-*OsTIR* and P$_{OpTEF1}$-*TetR'-OpSSN6-5flag* were grown in SD medium supplemented with appropriate nucleotide and amino acids in the presence of doxycycline at the indicated concentation. IAA was added at 0.5 mM and incubated at 30 °C for 2.5 h. Total cell extracts were prepared and subjected to a western analysis using anti-flag antibody. Intensity of bands was quantified with ImageJ software. In the presence of IAA and doxycycline, the mAID-Cdc5-flag protein was depeled by ~80%.

Figure S5

Result of pblast for *O. olymorpha* model proteins

Query: ScCdc15

	Protein ID	Location	Score	EValue	%Indt.	%Subj.Cov.	Sc homologue	gene name (this study)
1	52717	scaffold_7:244619-245632	513	1.47E-040	40.4	75.7	*ScBCK1*	*OpBCK1*
2	17280	scaffold_6:890165-892861	508	3.40E-048	42.2	50.4	-	*OpHCD1*
3	23749	scaffold_3:338716-340286	488	3.01E-37	48.7	39.2	-	*OpHCD2*
4	16151	scaffold_3:565100-568382	445	1.14E-039	43.9	28.9	*ScSTE11*	*OpSTE11*
5	15344	scaffold_1:1061265-1064094	439	7.34E-039	38.1	31.2	*ScSTE20*	*OpSTE20*
6	52579	scaffold_7:581327-582064	438	1.36E-034	47.3	68.7	*ScKIC1*	*OpKIC1*

Figure S5. Result of BLAST search using blastp for *O. polymorpha* model proteins at the genome portal of the Department of Energy Joint Genome Institute (https://mycocosm.jgi.doe.gov/Hanpo2/Hanpo2.home.html) [6]

Figure S6

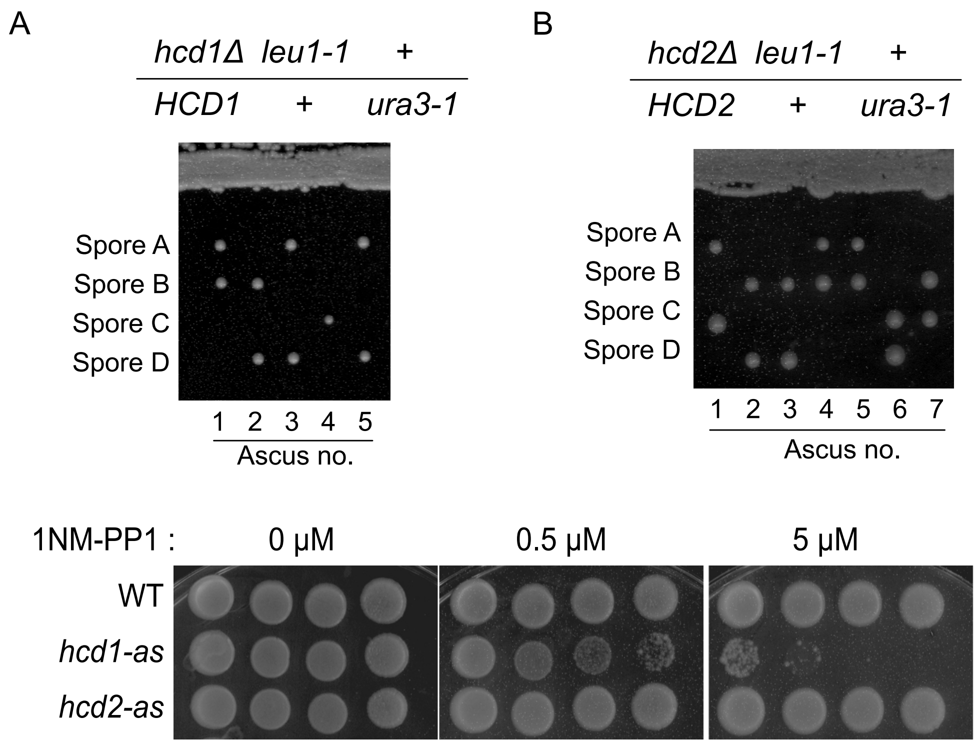

Figure S6 *OpHCD1* and *OpHCD2* are essential for growth

(A) Tetrad analysis of heterozygous *hcd1Δ*/+ diploid cells (HPH1834). Cells were incubated on an MAME sporulation plate before dissection of spores. Two or fewer colonies were formed in all tetrads.

(B) Tetrad analysis of heterozygous *hcd2Δ*/+ diploid cells (HPH2251). Cells were incubated on an MAME sporulation plate before dissection of spores. Two or fewer colonies were formed in all tetrads.

(C) Growth assay of the *hcd1-as* and *hcd2-as* mutants. Serial dilutions of wild type (HPH954), *hcd1-as* (HPH1894), and *hcd2-as* (HPH1909) strains were spotted on YPDS agar plates containing the indicated concentration of 1NM-PP1 and incubated at 30 °C.

Figure S7

A

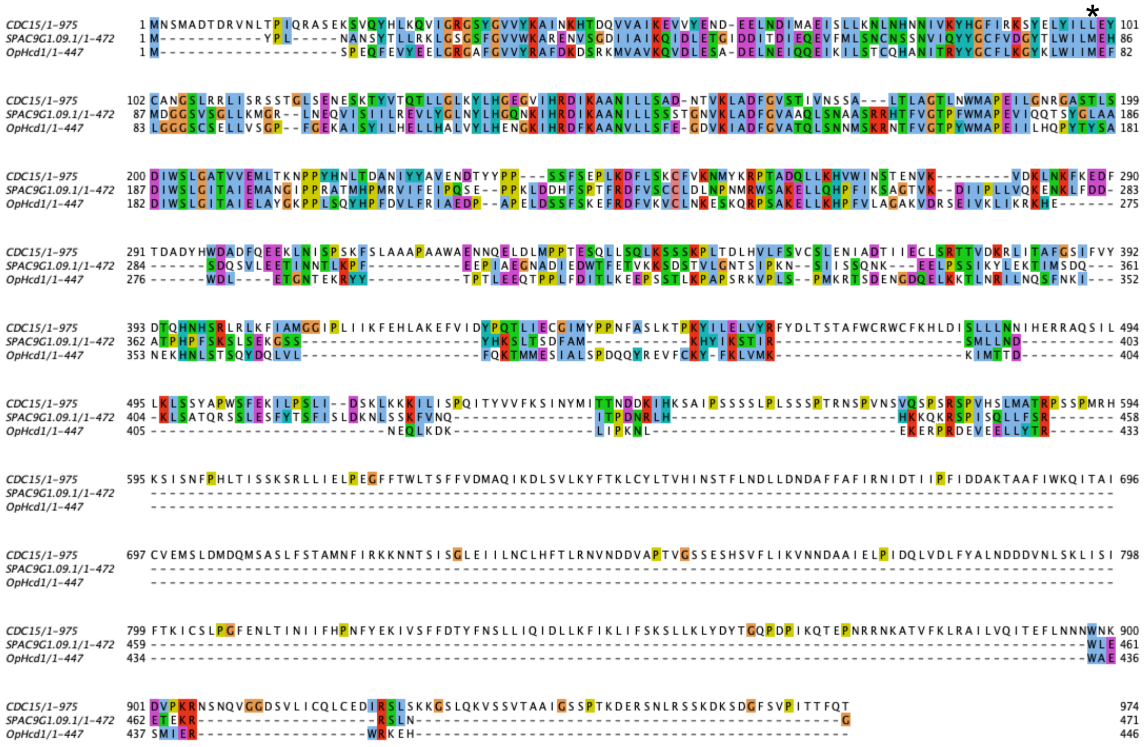

B

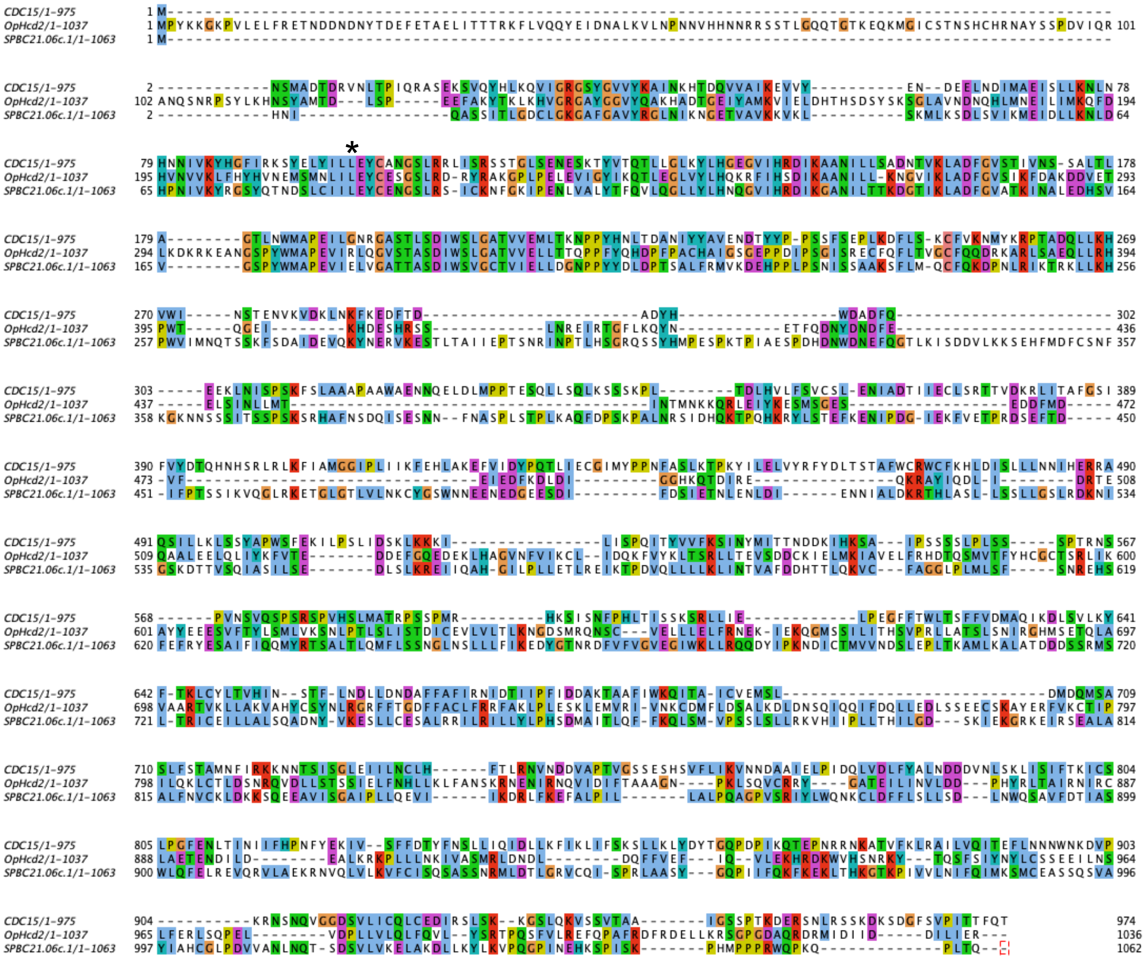

Figure S7. Amino acid alignments of OpHcd1, OpHcd2, ScCdc15, SpCdc7, and SpSid1. Amino acid sequences of OpHcd1, ScCdc15, and SpSid1 (A) or OpHcd2, ScCdc15, and SpCdc7 (B) were aligned with MafftWS by Jalview 2.8.2 [54]. Asterisks indicate the position of the amino acid of ScCdc15 whose mutation to glycine caused the ATP-analog 1NM-PP1 sensitivity.

Figure S8

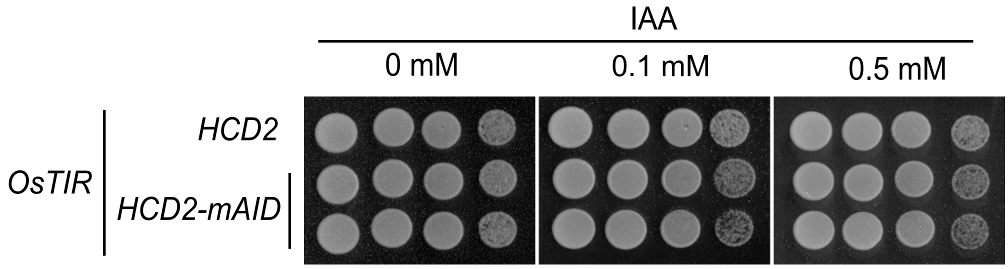

Figure S8. Growth assay of the *hcd2-mAID* mutant.
Serial dilutions of wild type (HPH1319), *hcd2-mAID* (HPH1599, HPH1600) strains were spotted on YPDS agar plates containing the indicated concentration of IAA and incubated at 30 °C.

Figure S9

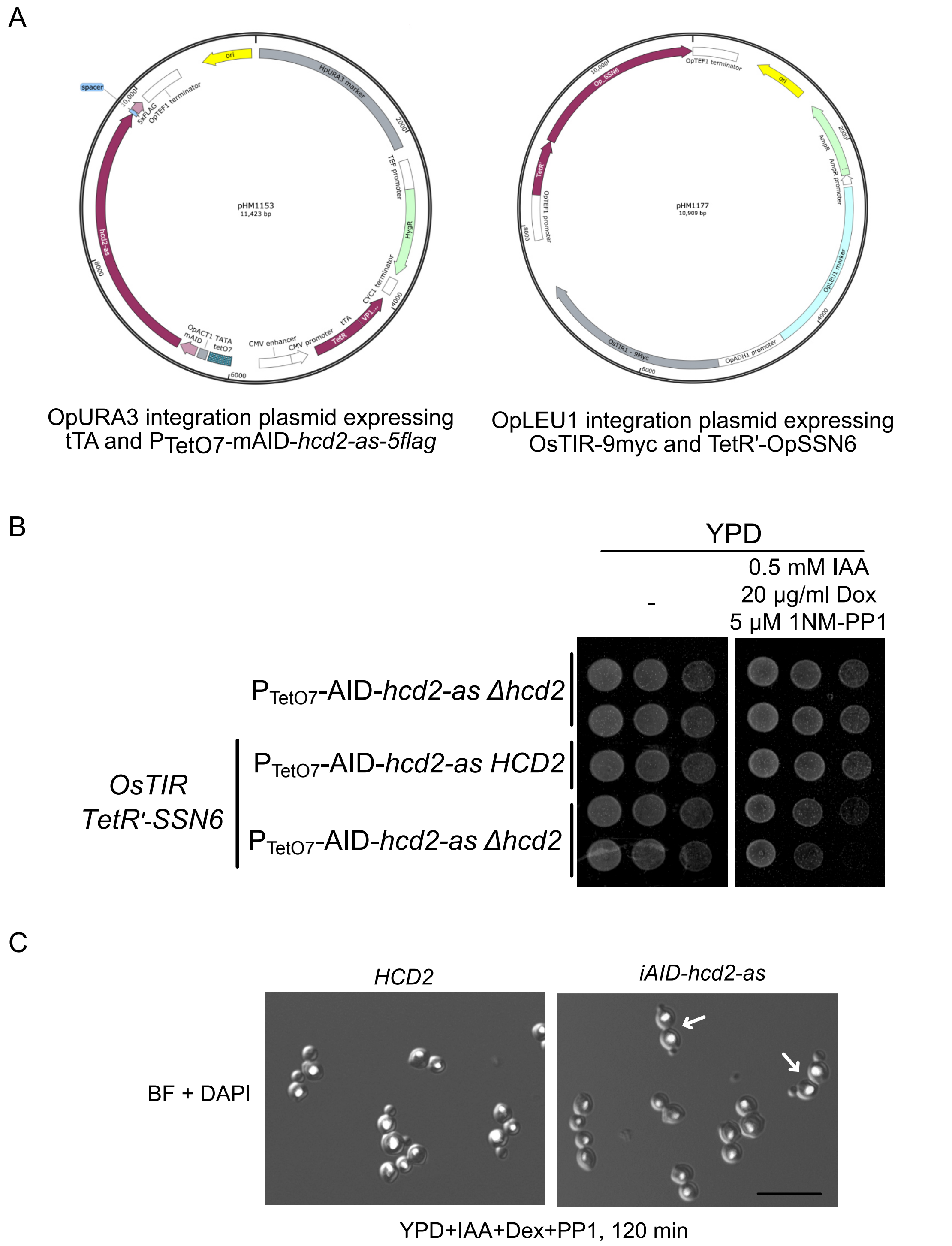

Figure S9 Phenotype of the iAID^{Op}-*hcd2-as* mutant

(A) Schematics of the plasmids used to construct the iAID^{Op}-*hcd2-as* mutant in the *hcd2Δ* background. The pHM1153 and pHM1177 were integrated into *OpURA3* and *OpLEU1* loci, respectively. The maps were drawn with SnapGene.

(B) Growth assay of the iAID^{Op}-*hcd2-as* mutant. Serial dilutions of strains with the indicated genotype were spotted on a YPDS agar plate and a YPDS plate containing 0.5 mM IAA, 20 μg/mL doxycycline, and 5μM 1NM-PP1 and incubated at 30 °C for 1day. Yeast strains: HPH2194, HPH2195, HPH2196, HPH2197, HPH2198.

(C) DAPI staining of iAID^{Op}-*hcd2-as* strains. Wild type (HPH2176) and iAID^{Op}-*hcd2-as* cells (HPH2105) carrying P$_{ADH1}$-*OsTIR*, P$_{CMV}$-tTA, P$_{OpTEF1}$-*TetR'-OpSSN6-5flag* as well as *hcd2-a*s cells (HPH1870) were grown in YPDS medium until logarithmical growth phase. Doxycycline, IAA, and 1NM-PP1 were added at 5 μg/mL, 0.5 mM, and 5 μM, respectively, and incubated at 30 °C for 2 h. Cells were fixed with 70 % ethanol and DNA was stained with DAPI. Bright field image and DAPI image were merged. Scale bar, 10 μm. Arrows indicate failure of cell separation in the previous mitosis.

Figure S10

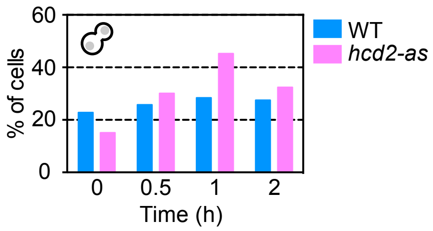

Figure S10. *hcd2-as* cells transiently accumulated late mitotic cells after addition of 1NM-PP1 Logarithmically growing wild type (HPH2186) and *hcd2-a*s (HPH1870) cells in YPDS medium were incubated with 5 μM 1NM-PP1 at 30 °C. Cells were fixed with 70% ethanol and DNA was stained with DAPI. The graph shows the percentage of large budded cells with two nuclei, one in the mother and the other in the bud, which represents the late mitotic stage. More than 100 cells were analysed at each time point.

Table S1 Yeast strains and plasmids

Name	Genotype/construction	Source
**Yeast strains**		
HPH656	ku80Δ::zeo leu1-1 ura3-1	lab stock
HPH951	ku80Δ::hphNT1 ade12-cr3 ura3-1	lab stock
HPH1047	wild type	lab stock
HPH1319	ku80Δ::zeo ura3-1<<P_{TEF1}-OsTIR-URA3	lab stock
HPH1497	CDC14-GFP-hphNT1 Δku80::zeo ura3-1	This study
HPH1599	HCD2-3mAID-natNT2 ku80Δ::zeo ura3-1<<P_{TEF1}-OsTIR-URA3	This study
HPH1600	HCD2-3mAID-natNT2 ku80Δ::zeo ura3-1<<P_{TEF1}-OsTIR-URA3	This study
HPH1605	HCD1-GFP-hphNT1 MPS3-mRFP-kanMX6 ura3-1	This study
HPH1608	HCD2-GFP-hphNT1 MPS3-mRFP-kanMX6	This study
HPH1714	HCD2-GFP-hphNT1 MPS3-mRFP-kanMX6 Δbub2::natNT2	This study
HPH1719	CDC14-GFP-hphNT1 CDC5-3mAID-natNT2 Δku80::zeo ura3-1<<TEF1promoter-OsTIR-URA3	This study
HPH1721	CDC14-GFP-hphNT1 TEM1-3mAID-natNT2 Δku80::zeo ura3-1<<TEF1promoter-OsTIR-URA3	This study
HPH1737	hcd1Δ::hphNT1/+ ku80Δ::zeo/ku80Δ::zeo leu1-1/+ +/ura3-1	This study
HPH1738	hcd2Δ::natNT2/+ ku80Δ::zeo/ku80Δ::zeo leu1-1/+ +/ura3-1	This study
HPH1761	BFA1-GFP-hphNT1 MPS3-mRFP-kanMX6 ade12-cr3	This study
HPH1769	HCD2-GFP-hphNT1 CDC5-3mAID-natNT2 Δku80::zeo ura3-1<<TEF1promoter-OsTIR-URA3 MPS3::HpMPS3-mRFP-HpkanMX	This study
HPH1834	hcd1Δ::hphNT1/+ ku80Δ::zeo/ku80Δ::zeo leu1-1/+ +/ura3-1 pHM898(Ku80+-OpKanMX6)	This study
HPH1870	hcd2Δ::natNT2<<Ophcd2-as(L215G)-hphNT1(pHM1121) ku80Δ::zeo ura3-1 pHM898(Ku80+-OpKanMX6)	This study
HPH1894	hcd1Δ::hphNT1<<Ophcd1-as(M80G)-natNT2(pHM1119)	This study
HPH1909	hcd2Δ::natNT2<<Ophcd2-as(L215G)-hphNT1(pHM1121) ku80Δ::zeo	This study
HPH1926	ura3-1::URA3-OpTEF1pro-TetR'-OpSSN6(pHM1129) ku80Δ::zeo	This study
HPH1942	ku80Δ::zeo leu1-1 ura3-1::pHM1132	This study
HPH1943	ku80Δ::zeo leu1-1 ura3-1::pHM1133	This study
HPH1956	HCD2-GFP-hphNT1MPS3-mRFP-kanMX6 TEM1<<TEM1(E70L)-5flag-natNT2	This study
HPH1968	GFP-TUB1-OpKanMX (HPH1047)	This study
HPH1969	hcd1Δ::hphNT1<<Ophcd1-as(M80G)-natNT2(pHM1119) GFP-TUB1-OpKanMX(pHM933)	This study
HPH2004	ura3-1::URA3-OpTEF1pro-TetR'-OpSSN6(pHM1129)::pHM1133 ku80Δ::zeo	This study
HPH2008	ura3-1ura3-1::URA3-OpTEF1pro-TetR'-OpSSN6(pHM1129)::pHM1143 ku80Δ::zeo	This study
HPH2009	ura3-1::URA3-OpTEF1pro-TetR'-OpSSN6(pHM1129)::pHM1145 Δku80::zeo	This study
HPH2042	ku80Δ::zeo ura3-1 ade12-cr3::P_{ADH1}-OsTIR-9myc-natNT2(pHM1147) TEF1::OpTEF1pro-TetR'-OpSSN6-URA3 (pHM1129)	This study
HPH2044	ku70Δ::natNT2 ura3-1::P_{tetO7}-CDC5-5flag-URA3(pHM1141) leu1-1	This study
HPH2046	ku70Δ::natNT2 ura3-::<P_{tetO7}-mAID-CDC5-5flag-URA3(pHM1152) leu1-1	This study
HPH2049	(ku70Δ::natNT2 ura3-1::P_{tetO7}-CDC5-5flag-URA3(pHM1141) leu1-1) / (ku80Δ::hphNT1 ura3-1 ade12-cr3::pHM1147 TEF1::pHM1129), diploid	This study
HPH2053	(ku70Δ::natNT2 ura3-1::P_{tetO7}-mAID-CDC5-5flag-URA3(pHM1152) leu1-1) x (ku80Δ::hphNT1 ura3-1 ade12-cr3::pHM1147 TEF1::pHM1129), diploid	This study
HPH2067	Δhcd2::natNT2 ku80Δ::zeo URA3::P_{tetO7}-mAID-hcd2-as-hphNT1-URA3(pHM1153) leu1-1 Ku80+-OpKanMX (pHM898)	This study

HPH2105	ku80Δ::zeo ura3-1 ade12-cr3::pHM1147 TEF1::pHM1129	This study
HPH2108	MOB1-GFP-hphNT1 MPS3-mRFP-OpKanMx(pHM968-1) Δku80::zeo ura3-1	This study
HPH2194	ku80Δ::zeo Δhcd2::natNT2 URA3::P_{tetO7}-mAID-hcd2-as-hphNT1-URA3(pHM1153) leu1-1<<ADH1p-OsTIR-TEF1p-TetR'-Ssn6-OpLEU1(pHM1177) Ku80+-OpKanMX (pHM898)	This study
HPH2195	ku80Δ::zeo Δhcd2::natNT2 URA3::P_{tetO7}-mAID-hcd2-as-hphNT1-URA3(pHM1153) leu1-1::P_{ADH1}-OsTIR, P_{TEF1}-TetR'-Ssn6-OpLEU1(pHM1177) Ku80+-OpKanMX (pHM898)	This study
HPH2196	ku80Δ::zeo HCD2 URA3::P_{tetO7}-mAID-hcd2-as-hphNT1-URA3(pHM1153) leu1-1::P_{ADH1}-OsTIR, P_{TEF1}-TetR'-Ssn6-OpLEU1(pHM1177)) Ku80+-OpKanMX (pHM898)	This study
HPH2197	ku80Δ::zeo Δhcd2::natNT2 URA3::P_{tetO7}-mAID-hcd2-as-hphNT1-URA3(pHM1153) Ku80+-OpKanMX (pHM898)	This study
HPH2198	ku80Δ::zeo Δhcd2::natNT2 URA3::P_{tetO7}-mAID-hcd2-as-hphNT1-URA3(pHM1153) Ku80+-OpKanMX (pHM898)	This study
HPH2244	ku80Δ::zeo Δhcd2::natNT2 URA3::P_{tetO7}-mAID-hcd2-as-hphNT1-URA3(pHM1153) leu1-1::P_{ADH1}-OsTIR, P_{TEF1}-TetR'-Ssn6 (pHM1198-1) Ku80+-OpKanMX (pHM898)	This study
HPH2245	ku80Δ::zeo Δhcd2::natNT2 URA3::P_{tetO7}-mAID-hcd2-as-hphNT1-URA3(pHM1153) leu1-1::P_{ADH1}-OsTIR(F74A), P_{TEF1}-TetR'-Ssn6 (pHM1200) Ku80+-OpKanMX (pHM898)	This study
HPH2246	ku80Δ::zeo leu1-1::P_{ADH1}-OsTIR(F47G), P_{TEF1}-TetR'-Ssn6, LEU1(pHM1198-2) ura3-1	This study
HPH2247	ku80Δ::zeo leu1-1::P_{ADH1}-OsTIR(F47A), P_{TEF1}-TetR'-Ssn6 LEU1 (pHM1198-2) ura3-1	This study
HPH2251	hcd2Δ::natNT2/+ ku80Δ::zeo/ku80Δ::zeo leu1-1/+ +/ura3-1 Ku80+-OpKanMX (pHM898), diploid	This study
HPH2254	ku80Δ::zeo leu1-1::P_{ADH1}-OsTIR, P_{TEF1}-TetR'-Ssn6, LEU1 (pHM1198-1) ura3-1	This study
HPH2258	ku80Δ::zeo leu1-1::P_{ADH1}-OsTIR(F47A), P_{TEF1}-TetR'-Ssn6(pHM1198-2) TUB1::pHM1136 ura3-1	This study
HPH2260	ku80Δ::zeo Δhcd2::natNT2 URA3::P_{tetO7}-mAID-hcd2-as-hphNT1-URA3(pHM1153) ade12-cr3::P_{ADH1}-OsTIR(F74A), P_{TEF1}-TetR'-Ssn6 (pHM1201) TUB1::pHM1136	This study
HPH2270	ku80Δ::zeo Δhcd2::natNT2 URA3::P_{tetO7}-mAID-hcd2-as-hphNT1-URA3(pHM1153) leu1-1::P_{ADH1}-OsTIR, P_{TEF1}-TetR'-Ssn6 (pHM1198-1) Ku80+-OpKanMX (pHM898)	This study

plasmids

pHM898	OpURA3-hphNT1-based integration plasmid carrying OpKU80+	This study
pHM933	pFA6a plasmid carrying P_{OpTEF1}-kanMX6 and P_{OpTUB1}-GFP-OpTUB1	This study
pHM1119	pFA6a-natNT2 plasmid carrying Ophcd1^{M80G}	This study
pHM1121	pFA6a-hphNT1 plasmid carrying Ophcd2^{L215G}	This study
pHM1129	pFA6a-hphNT1 plasmid carrying TetR'-OpSSN6	This study
pHM1133	OpURA3-hphNT1-based integration plasmid carrying rtTA, P_{tetO7}-CDC5-5flag	This study
pHM1136	pFA6a plasmid carrying P_{OpTEF1}-kanMX6, P_{OpTUB1}-GFP-OpTUB1, histoneH3-mCherry-histoneH4	This study
pHM1141	OpURA3-hphNT1-based integration plasmid carrying rtTA, P_{tetO7}-CDC5-5flag-URA3	This study
pHM1143	OpURA3-hphNT1-based integration plasmid carrying rtTA, P_{tetO7}-SPC72-5flag-URA3	This study
pHM1145	OpURA3-hphNT1-based integration plasmid carrying rtTA, P_{tetO7}-GFP-5flag-URA3	This study
pHM1147	natNT2 based integration plasmid carrying the N-terminal and upstream fragment of OpADE12, P_{ADH1}-OsTIR-9myc-natNT2(pHM1147)	This study
pHM1152	OpURA3-hphNT1-based integration plasmid carrying rtTA, P_{tetO7}-mAID-CDC5-5flag	This study
pHM1153	OpURA3-hphNT1-based integration plasmid carrying rtTA, P_{tetO7}-mAID-hcd2^{L215G}-5flag	This study
pHM1177	OpLEU1-based integration plamid carrying P_{ADH1}-OsTIR, P_{TEF1}-TetR'-OpSSN6	This study
pHM1198-1	OpLEU1-based integration plamid carrying P_{ADH1}-OsTIR, P_{TEF1}-TetR'-OpSSN6	This study
pHM1198-2	OpLEU1-based integration plamid carrying P_{ADH1}-OsTIR(F74G), P_{TEF1}-TetR'-OpSSN6	This study
pHM1200	OpLEU1-based integration plamid carrying P_{ADH1}-OsTIR(F74A), P_{TEF1}-TetR'-OpSSN6	This study
pHM1201	OpADE12-based integration plamid carrying P_{ADH1}-OsTIR(F74A), P_{TEF1}-TetR'-OpSSN6	This study

Table S2. iAID plasmids for *O. polymorpha*

Plasmid	pHM1141

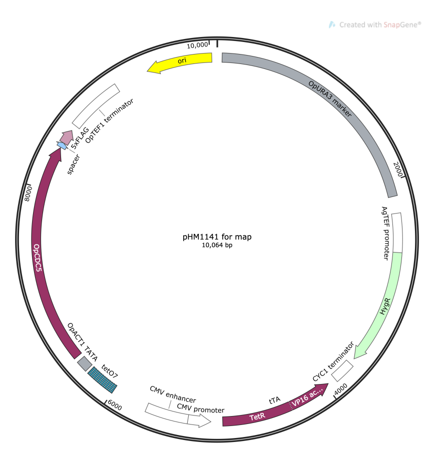

OpURA3 marker: 43..2135
hphNT1: 2287..3902
CMVenhancer,promoter-tTA: 5670..4982
tetO7:5999..6269
OpACT1 TATA: 6294..6398
OpCDC5: 6437..8393
Spacer-5xFLAG: 8399..8575
OpTEF1 terminator: 8629..9128

ACGCGGCCGCCAGCTGAAGCTTCGTACGCTGCAGGTCGACGGATCTCCAGAACCTGCTACACCATCTACGCCAGCAAGTGGAGATGAAGAACGTGGGAGCAAGCGACGCGGA[illegible]

Other plasmids: pHM1143, pHM1145
OpSPC72 or GFP are inserted in place of OpCDC5 in pHM1143 and pHM1145, respectively.

Plasmid	pHM1153

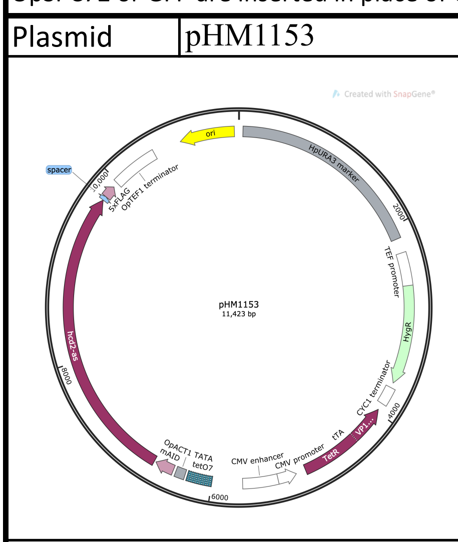

OpURA3 marker: 43..2135
hphNT1: 2287..3902
CMVenhancer,promoter-tTA: 5670..4982
tetO7:5999..6269
OpACT1 TATA: 6294..6398
mAID: 6428..6631
hcd2-as: 6650..9757
Spacer-5xFLAG: 9758..9802
OpTEF1 terminator: 9988..10487

[illegible]

Other plasmids: pHM1152
OpCDC5 is inserted in place of hcd2-as in pHM1152.

Plasmid	pHM1177, pHM1198-1

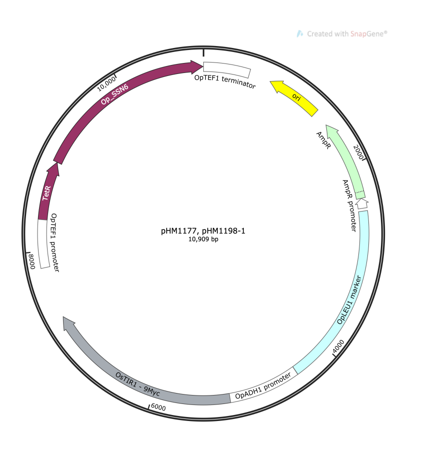

OpTEF1 terminator: 4..503
OpLEU1 marker: 2495..4399
OpADH1 promoter: 4402..5166
OsTIR-9myc: 5167..7278
OpTEF1 promoter: 7827..8326
TetR': 8327..8947
OpSSN6: 8948..10906

[illegible]

Other plasmids: pHM1198-2, pHM1200
OsTIR (F74G)-9myc or OsTIR (F74A)-9myc is inserted in place of OsTIR-9myc in pHM1198-2 and pHM1200.

Plasmid	pHM1201

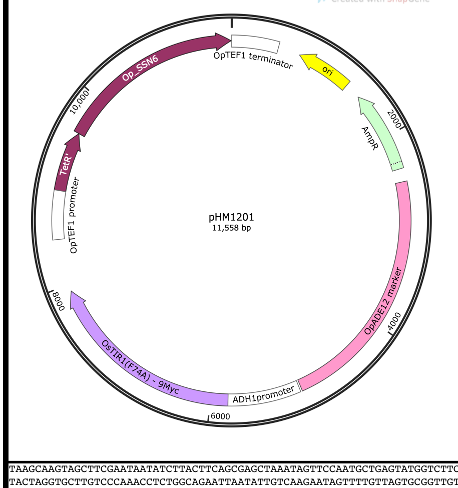

OpTEF1 terminator: 4..503
OpADE12 marker: 2495..5032
OpADH1 promoter: 5051..5815
OsTIR(F74A)-9myc: 5816..7927
OpTEF1 promoter: 8494..8975
TetR': 8976..9596
OpSSN6: 9597..11555

[illegible]
